# Illinois State Medical Society

**Published:** 1860-09

**Authors:** E. L. Holmes

**Affiliations:** Chicago, Box 2175


### ILLINOIS STATE MEDICAL SOCIETY.

The subscriber respectfully requests the members of this Society, and the profession at large, to assist in accomplishing the object for which the Special Committee on Diseases of the Eye was appointed.

Reports of ophthalmic cases and papers upon any subject in this department of medical science, are solicited.

The Committee particularly request papers upon "The peculiar causes of conjunctival inflammation, in different parts of Illinois and the adjoining States; as also the history of cases of sympathetic ophthalmitis in one eye following injuries and chronic inflammation of the iris and choroid of the other eye."

It is hoped the profession generally will take interest in this important subject, and forward all communications to the Committee before April 1, 1861.

E. L. Holmes,
Chicago, Box 2175.

